# Cardiac magnetic resonance imaging of systemic amyloidosis patients with normal left ventricular ejection fraction: An initial study

**DOI:** 10.12669/pjms.296.3775

**Published:** 2013

**Authors:** Rui Xia, Fabao Gao, Jiayu Sun, Chunchao Xia, Zhangxue Hu, Yingkun Guo

**Affiliations:** 1 Rui Xia, PhD, Resident Doctor, Department of Radiology, West China Hospital, Sichuan University, No. 37 Guoxuexiang, 610041 Chengdu, China.; 2 Fabao Gao, MD PhD, Professor, Department of Radiology, West China Hospital, Sichuan University, No. 37 Guoxuexiang, 610041 Chengdu, China.; 3 Jiayu Sun, PhD, Supervising Technician, Department of Radiology, West China Hospital, Sichuan University, No. 37 Guoxuexiang, 610041 Chengdu, China.; 4 Chunchao Xia, BS, Technician, Department of Radiology, West China Hospital, Sichuan University, No. 37 Guoxuexiang, 610041 Chengdu, China.; 5 Zhangxue Hu, MD PhD, Professor, Department of Nephrology, West China Hospital, Sichuan University, No. 37 Guoxuexiang, 610041 Chengdu, China.; 6 Yingkun Guo, MD PhD, Associate Professor, Department of Radiology, West China Second University Hospital, Sichuan University, No. 20 Sec 3 Renmin Road South, 610041 Chengdu, China.

**Keywords:** Myocardial amyloidosis, Systemic amyloidosis, Magnetic resonance imaging, echocardiography, Late gadolinium enhancement

## Abstract

***Objective:*** The purpose of this study was to find whether Cardiac Magnetic Resonance (CMR) could assess the myocardial interstitium in patients suffering from systemic amyloidosis with normal left ventricular ejection fraction.

***Methods:*** *Twenty Six* patients in whom systemic amyloidosis was confirmed by kidney biopsy were investigated. Five patients with normal left ventricular ejection fraction were selected. The heart function of the patients was diagnosed by two-dimensional transthoracic echocardiography. The main MR sequences include an inversion recovery prepared echo planar imaging perfusion sequence, inversion recovery TrueFISP sequence (delayed enhancement) and TrueFISP cine sequence for heart function measurement (including ejection fraction (EF), end diastolic volume (EDV), end systolic volume (ESV), stroke volume (SV) and cardiac output (CO)).

***Results*** **:** Perfusion defects were seen in three patients. In these patients, myocardial enhancement was visible on late gadolinium enhancement images. The enhancement pattern was diffuse in three patients and focal in two patients. Heart dysfunction was mild, as follows: EF normal (range, 56-75%; mean, 69.4%), ESV normal (range, 15.7-30.0; mean, 23.0), EDV decreased (range, 42.1-96.6; mean, 72.7), SV decreased (range, 23.7-68.6; mean, 49.6) and CO normal (range, 2.6-5.9; mean, 3.9). Hematoxylin and eosin stain and Congo red stain demonstrated typical amyloid deposits. Amyloidosis was classified as amyloid light chain by kappa and lambda stain.

***Conclusions:*** Cardiac Magnetic Resonance could detect abnormal myocardial interstitium in systemic amyloidosis patients with normal left ventricular ejection fraction.

## INTRODUCTION

Amyloidosis are uncommon systemic diseases characterized by the extracellular deposition of pathologic insoluble amyloid protein in organs and tissues. Amyloidosis are usually classified into four major types: primary (systemic), familial, secondary and senile.^1^ Systemic amyloidosis involves deposition of the immunoglobulin light chain. Amyloid light chain (AL) is the most common pathologic subtype of amyloidosis. AL amyloidosis is usually caused by a smouldering clonal plasma cell disorder producing monoclonal light chains that undergo conformational modifications and aggregate into amyloid fibrils.^2^^,^^3^ Amyloid fibril deposits commonly affect the kidneys, heart, liver and peripheral and autonomic nervous systems.^4^ Cardiac involvement is common in AL amyloidosis and is associated with various treatment options and prognoses.^5^^-^^7^ In AL amyloidosis, the heart is affected in nearly 50% of cases.^6^ Previous reports indicated cardiac involvement as the cause of death in approximately 50% of patients with AL amyloidosis.^8^^-^^10^

Assessment of cardiac function is important in the diagnostic work-up, therapeutic follow-up and prognosis of AL amyloidosis, as well as in the clinical management of these patients. Currently, myocardial biopsy is regarded as the gold standard test for diagnosis of cardiac involvement in AL amyloidosis.^2^ In clinical practice, however, the diagnosis of cardiac amyloidosis is usually made by echocardiography^7^, electrocardiography (ECG)^8^ and scintigraphy^8^ with the aid of a radiolabelled serum amyloid P component so as to avoid invasive and problematic biopsy. According to previous reports, cardiac involvement in diagnosis of AL amyloidosis by these modalities has some limitations (Echocardiographic features are not highly speciﬁc individually, especially when hypertrophy from other causes is present. Electrocardiographic does not correlate with survival, and can’t supply heart function parameters. Planar Radiolabeled serum amyloid P component (SAP) scintigraphy is unable to image amyloid in the moving heart.).^11^^-^^13^

Cardiac magnetic resonance (CMR) has been reported as a promising modality in the evaluation of suspected amyloid heart disease, especially using the late gadolinium enhancement (LGE) technique to detect significant findings indicating cardiac involvement in AL amyloidosis.^5^^,^^14^ Now the ability of CMR detecting early cardiac involvement caused by AL amyloidosis is unknown. Although the condition is extremely rare, herein we investigate the diagnostic value of CMR in AL amyloidosis patients with normal left ventricular (LV) eject fraction (EF) on echocardiography.

## METHODS

We retrospectively reviewed 26 patients (age range, 38-72 years) with documented amyloidosis by kidney biopsy at the inpatient clinic of West China Hospital. Then the heart function of the patients was assessed by two-dimensional transthoracic echocardiography. Exclusion criteria included myocardial ischemia, myocarditis. Five patients with normal LVEF were recruited while 21 patients were excluded by the criteria.

Five patients (4 men; 1 woman; age range, 42-61 years) with normal left ventricular ejection fraction were chosen by two-dimensional transthoracic echocardiography. All of these 5 patients suffered from proteinuria, puffiness around the eyes or pitting oedema of the legs. And nephrotic syndrome was diagnosed according to the measurement of 24-h urinary protein level.

Our study was approved by the Ethics Committee of West China Hospital of Sichuan University. Informed consent was obtained from each of the patients for echocardiography and MRI.

***Histopathology: ***All patients underwent renal biopsy. Histopathological methods include hematoxylin and eosin (HE), Congo red, kappa and lambda staining. Electron microscopy was also performed for all the patients.

***Echocardiography: ***About 10 days after renal biopsy, Two-dimensional transthoracic echocardiography and colour Doppler flow imaging were performed in all patients using a phased array 2-5-MHz transducer (iE33; Philips Medical Systems, Andover, MA, USA) according to the guidelines of the American Society of Echocardiography.^10^

The following parameters were evaluated: bi-ventricular and bi-atrial internal diameter, LV wall thickness and inter-ventricular and atrial septal thickness. All morphological parameters were measured at end diastole. LV function parameters were calculated by Simpson’s modified bi-plane method.

***Cardiac Magnetic Resonance Imaging: ***On the day of echocardiography examination, CMR imaging was performed with a 3.0-T whole-body MR system (MAGNETOM Trio, Siemens Medical Solutions, Erlangen, Germany) for all the patients. Data was acquired during end-inspiratory breath holding. After scout images, TrueFISP cine sequence (TR/TE, 44.9/2.4 msec; field of view (FOV), 340 × 276 mm; matrix size, 192 × 125; flip angle, 50°; slice thickness, 9 mm) with retrospective ECG-gating was used to acquire dynamic cine loops of the LV for function analysis. The LV was imaged in its entirety from the base to the apex in 9-12 short-axis cine images without inter-slice gaps or overlaps.

First-pass myocardial perfusion imaging was performed using an inversion recovery prepared echo planar imaging sequence (TR/TE, 200/1.1 msec; TI, 100ms; FOV, 360 × 270 mm; matrix size, 256 × 205; slice thickness, 6 mm, 3 short axis slices and 1slice 4 chamber view). All patients received 0.1 mmol/kg bodyweight gadolinium-diethylene-triamine penta-acetic acid (DTPA; Magnevist, Schering, Berlin, Germany) through an intravenous ante-cubital catheter infused by an automated injector (Stellant, MEDRAD, Indianola, PA, USA) at a flow rate of 2.0 ml/sec. Contrast injection was followed by a saline bolus chaser of 20 ml at the same flow rate. LGE images were obtained using an inversion recovery TrueFISP sequence with retrospective ECG-gating 10-15 minutes after administration of a second gadolinium-DTPA bolus (0.05 mmol/kg at a flow rate of 1.0 ml/sec; TR/TE 744/1.28 msec; FOV, 300 × 272 mm; matrix size, 256 × 184; slice thickness, 6 mm). TIs for LGE were selected according to TI Scout which was performed before LGE.

Cine function analysis was performed off-line with commercial software (Argus, Siemens Medical Solutions) by two experienced radiologists blinded to the echocardiograph results. In every short-axis slice, endocardial and epicardial boundaries were traced semi-automatically during the end-diastolic and end-systolic phases in order to obtain the volumetric parameters of LV (end diastolic volume [EDV], end systolic volume [ESV], stroke volume [SV], and ejection fraction [EF]) via the section summation method. Two experienced radiologists analyzed and interpreted first-pass myocardial perfusion and LGE images. Discrepancies in their interpretations were resolved by consensus.

***Statistical Analysis: ***Paired-Samples T Test was used to compare heart functional parameters **(EDV, ESV, SV, EF)** measured by ultrasound and MRI. Statistical analysis was performed using SPSS 13.0 (SPSS Inc., Chicago, III, USA).

## RESULTS

**Fig.1:**
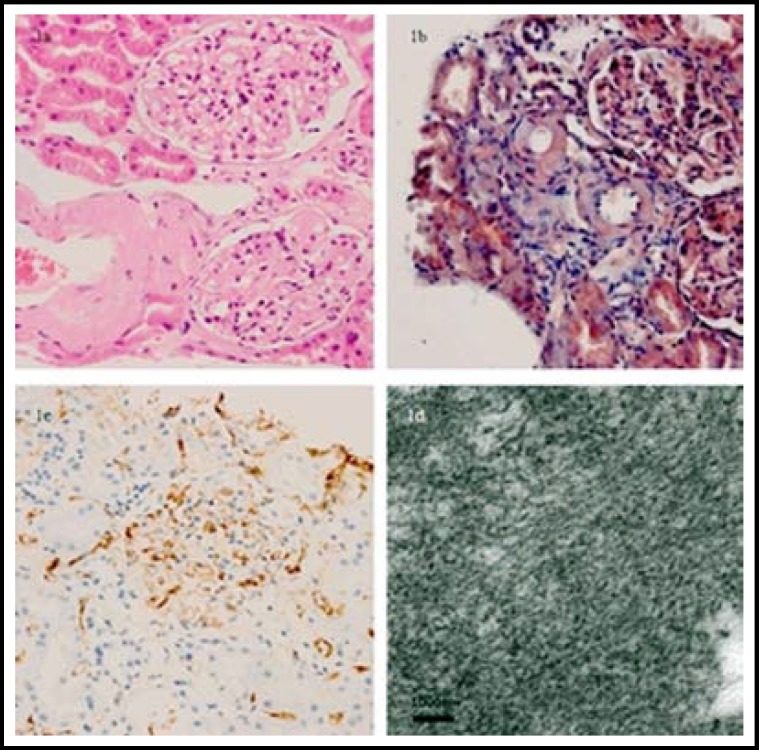
Histopathological findings of the renal biopsy in a 46-year-old man with systemic amyloidosis. Amyloid deposition in the interstitial of kidney demonstrated (a) red by HE staining (×200) and (b) orange by Congo red staining (× 200). The interstitial material is positively stained by Lambda staining and showed (c) brown (× 200). (d) Deposits composed of nonbranching fibrils were showed under the electron microscopy (× 8000).

***Histopathological Results: ***HE and Congo red stain demonstrated a typical amyloid deposit. Amyloidosis was classified as AL by kappa and lambda stain (kappa stain was negative while lambda stain was positive). Deposits composed of non-branching fibrils were viewed by electron microscopy (Fig.1).

**Fig.2:**
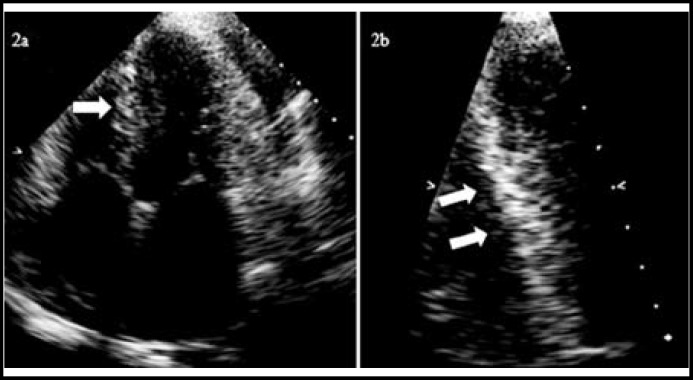
Echocardiography in a 57-year-old man with systemic amyloidosis. “Sparkling” appearance (white arrows) of ventricular septal wall showed in (a) four-chamber view and (b) ventricular septal wall view

**Fig.3:**
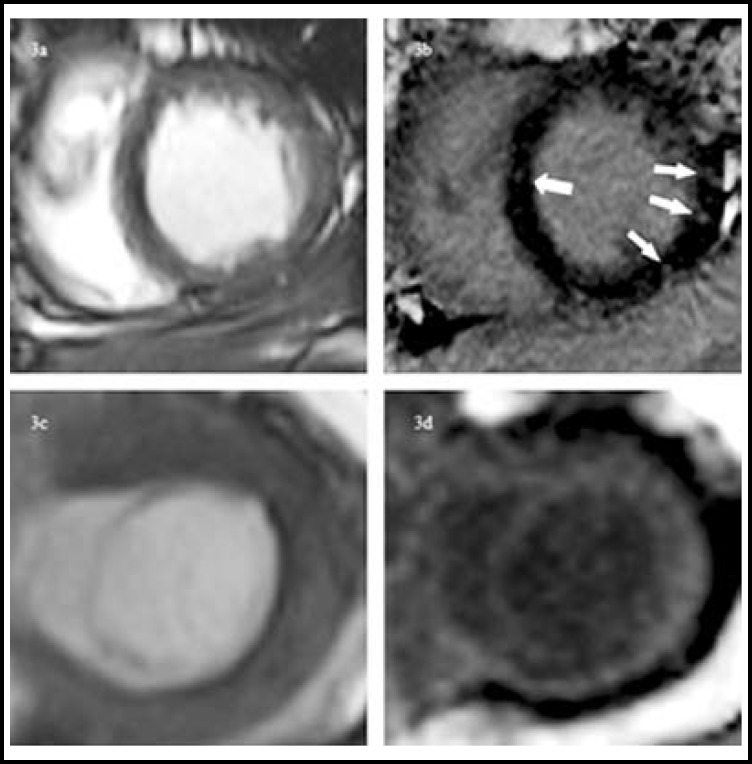
MR findings in 2 patients with systemic amyloidosis. Cine MRI (a, c) and LGE-MRI (b, d) in the cardiac short-axis plane. Image of 46-year-old man showed (b) focal delay enhancement (white arrows) and. Image of 57-year-old man showed (d) diffuse delay enhancement

**Table-I:** The baseline characteristics of systemic amyloidosis patients

*Patient number*	*1*	*2*	*3*	*4*	*5*	*Normal range*
Age	46	42	57	46	61	—
Sex	F	M	M	M	M	—
Echocardiography						
EF(%)	66	67	69	68	67	50-75
EDV(ml)	81	45	73	78	91	84-132
ESV(ml)	28	15	23	25	30	29-61
SV(ml)	53	30	50	53	61	35-90
MR						
EF(%)	74	75	56	69	73	56-78
EDV(ml)	96.6	62.7	42.1	73	88.9	77-195
ESV(ml)	28	15.7	18.5	23	30	19-72
SV(ml)	68.6	47	23.7	50	58.9	51-133
CO(L/min)	5.9	3.4	2.6	3.5	4.1	2.82-8.82

(EF ejection fraction, EDV end diastolic volume, ESV end systolic volume, SV stroke volume, CO cardiac output).

**Table-II:** CMR findings of systemic amyloidosis patients

*Patient number*	*1*	*2*	*3*	*4*	*5*
Septal thickness(mm)	16	19	17	13	14
Perfusion defect	—	—	present	present	Present
Focal enhancement	—	Present	—	present	—
Enhancement area	—	anterior	—	lateral, septal	—
Diffuse enhancement	Present	—	present	—	Present
Pleural effusions	Present	Present	Present	Present	Present
Pericardial effusions	Present	Present	Present	Present	Present

***Echocardiographic Findings: ***All of these patients showed enlarged left atrial internal diameter (range, 36-42 mm; mean, 39 mm; normal range, 20-35 mm) and increased LV wall thickness (>13 mm; normal range, 6-12 mm). 4 patients had depressed LV diastolic function (EDV: range, 45-91 ml; mean, 73.6 ml; normal range, 84-132 ml). EF was normal (EF: 67-69%; mean, 67.4%; normal range, 50-75%; Table-I). In one patient, characteristic granular sparkling appearance was evident in the septal wall. Pleural and pericardial effusion was found in all patients (Fig.2).

***CMR Features: ***Increased thickness of the LV septal wall was observed in all patients (diastolic septal thickness: range, 14-19 mm; mean, 15.8 mm; normal range, 7-11 mm). Systolic function (EF: range, 56-75%; mean, 69.4%) was normal. Perfusion defects were seen in 3 patients. Focal defects were located mainly in the anterior, lateral and septal myocardium. In all patients, the myocardium was enhanced on LGE images. The enhancement pattern was diffuse in three patients and focal in two patients. Focal areas of LV contrast enhancement were located mainly in the mid and basal segments of the anterior, lateral and septal myocardium. In all cases, CMR found pleural and pericardial effusions (Table-II, Fig.3).

No significant differences were found between heart functional parameters measured by Echocardiography and MRI (EF P=0.64, EDV P=0.92, ESV P=0.29, SV P=0.98).

## DISCUSSION

The results of this study demonstrated that cardiac MR imaging was comparable to echocardiography in accurately assessing the cardiac function of AL amyloidosis patients. Furthermore, perfusion defects were observed on first-pass myocardial perfusion images and various enhancements of the myocardium on LGE images in most patients. In patients with normal EF values, however, echocardiography detected abnormalities of the septum in only one patient.

In clinical settings, the diagnosis of cardiac involvement in AL amyloidosis is frequently made by echocardiography. As shown in our study, echocardiographic examination could quantitatively assess cardiac involvement via measurement of LV wall thickness, evaluation of restricted diastolic function and assessment of disproportionate atrial enlargement. These physiological changes indicate that deposition of amyloid fibrils in myocardial tissue results in reduced ventricular compliance, impairment of relaxation and eventually contraction.^13^^,^^15^ Of the patients with normal EF values in the present study, however, echocardiography subjectively evaluated myocardial abnormalities in only one case. While all patients suffered from nephrotic syndrome, none had clinical signs or symptoms of cardiac involvement. These findings indicated that echocardiography may be less sensitive for assessing mild cardiac involvement at the early clinical stage. Further studies with a larger sample of patients within normal EF ranges are needed to confirm these results.

Currently, CMR is considered the reference standard for non-invasive measurement of cardiac ventricular function and an important tool for accurate evaluation of morphologic features.^16^ Similar to previous reports, in this study, CMR without LGE technique detected the typical cardiac features of AL amyloidosis, including LV wall thickening, restriction of diastolic filling and disproportionate atrial enlargement. At present, no direct non-invasive method is available to assess and monitor the myocardial amyloid burden. Gadolinium CMR techniques may be an appealing method in the evaluation and diagnosis of suspected amyloid heart disease, especially using first-pass myocardial perfusion and LGE techniques. In our study, perfusion defects were observed in 3 of the 5 patients on first-pass myocardial perfusion scanning. As significant epicardial coronary artery disease was not present in these patients, these perfusion defects may result from either significant subendocardial deposition of amyloid protein or possible infiltration of small-calibre coronary vessel walls by amyloid.^17^^,^^18^

LGE has been used to evaluate cardiac amyloidosis and interstitial expansion due to amyloid deposition to some extent. Features of cardiac amyloidosis detectable by LGE-CMR have been well documented.^5^ The characteristics of cardiac amyloidosis on LGE images include focal (heterogeneous) and diffuse (homogeneous) enhancement, depending on the deposition and expansion of amyloid into the myocardium.^19^ In the cases described here, diffuse enhancement was observed in three patients and focal enhancement in two patients. A previous study reported MR findings in patients with mild global depression of systolic function (EF range, 41-54%; mean, 48.8%).^20^ However, special ability of MR to elucidate features of AL amyloidosis in patients with normal EF values has not been reported. In our study, myocardial abnormalities were detected in all patients using the LGE technique, but echocardiography detected only one case. Because cardiac involvement changes treatment options and adversely impacts prognosis, early detection is important for AL patients.^5^

Our study has several limitations. First, AL amyloidosis is extremely rare, only a limited number of patients were enrolled. Another limitation is that no patient underwent endomyocardial biopsy due to invasive injury.In summary, CMR techniques could detect earlier myocardium abnormalities in AL amyloidosis patients with normal EF.

## Notes

***Source of Funding:*** This study was supported by The State Key Program of National Natural Science Foundation of China (81130027) and The National Natural Science Foundation of China (30870687).
